# A Potential Quorum-Sensing Inhibitor for Bronchiectasis Therapy: Quercetin–Chitosan Nanoparticle Complex Exhibiting Superior Inhibition of Biofilm Formation and Swimming Motility of *Pseudomonas aeruginosa* to the Native Quercetin

**DOI:** 10.3390/ijms22041541

**Published:** 2021-02-03

**Authors:** The-Thien Tran, Kunn Hadinoto

**Affiliations:** School of Chemical and Biomedical Engineering, Nanyang Technological University, 62 Nanyang Drive, Singapore 637459, Singapore; tttran@ntu.edu.sg

**Keywords:** quercetin nanoparticles, drug-polyelectrolyte complexation, amorphous drugs, quorum sensing, bronchiectasis therapy, *Pseudomonas* biofilm

## Abstract

Quercetin (QUE)—a plant-derived flavonoid, is recently established as an effective quorum sensing (QS) inhibiting agent in *Pseudomonas aeruginosa*—the main bacterial pathogen in bronchiectasis lungs. Successful clinical application of QUE, however, is hindered by its low solubility in physiological fluids. Herein we developed a solubility enhancement strategy of QUE in the form of a stable amorphous nanoparticle complex (nanoplex) of QUE and chitosan (CHI), which was prepared by electrostatically driven complexation between ionized QUE molecules and oppositely charged CHI. At its optimal preparation condition, the QUE–CHI nanoplex exhibited a size of roughly 150 nm with a 25% QUE payload and 60% complexation efficiency. The complexation with CHI had no adverse effect on the antibacterial and anticancer activities of QUE, signifying the preservation of QUE’s bioactivities in the nanoplex. Compared to the native QUE, the QUE–CHI nanoplex exhibited superior QS inhibition in suppressing the QS-regulated swimming motility and biofilm formation of *P. aeruginosa*, but not in suppressing the virulence factor production. The superior inhibitions of the biofilm formation and swimming motility afforded by the nanoplex were attributed to (1) its higher kinetic solubility (5-times higher) that led to higher QUE exposures, and (2) the synergistic QS inhibition attributed to its CHI fraction.

## 1. Introduction

Chronic obstructive pulmonary disease (COPD) affects more than 300 million people worldwide, with an estimated mortality rate of 3 million people annually, which is caused primarily by tobacco smoking and environmental pollution [1,2]. COPD currently represents the third leading cause of death globally, behind only heart diseases and cancers, and it is expected to become the first leading cause of death in the next decade [3]. A majority of moderate to severe COPD patients suffer from bronchiectasis symptoms characterized by persistent infection and inflammation of the airways that leads to the gradual loss of lung function and eventually mortality [4,5].

Antibiotic therapy, preferably by the pulmonary delivery route, has been the mainstay in the long-term and intermittent treatments of COPD with bronchiectasis symptoms, together with bronchial hygiene therapy and reduction of airway inflammation [6]. While the current approach of therapy is effective, the concern over the long-term use of inhaled antibiotics is that it will lead to the rise of antibiotic-resistant bacterial pathogens, resulting in worsened exacerbation of the disease [7,8]. For this reason, alternative treatment approaches that can break the vicious cycle of airways infection and inflammation have been actively investigated to complement antibiotic therapy [9].

*Pseudomonas aeruginosa* bacteria have been identified as the main pathogens responsible for severe infection leading to acute exacerbations in bronchiectasis lungs [10]. The predominant mode of growth of *P. aeruginosa* is in the form of biofilm colonies—sessile communities of bacterial cells enclosed by self-secreted matrices of extracellular polymeric substances (EPS) [11]. Owing to the EPS acting as a physical barrier, biofilm cells notoriously exhibit a significantly lower susceptibility towards antibiotics than their planktonic counterparts making biofilm difficult to eradicate [12].

Quorum sensing (QS)—a cell-to-cell communication mechanism that generates signaling molecules to coordinate gene expression—is known to play an important role in the biofilm formation of *P. aeruginosa* [13]. In addition, QS also regulates the gene expression responsible for the production of *P. aeruginosa* virulence factors and bacterial motility that is crucial for biofilm formation [14,15]. By secreting and sensing extracellular signals, QS enables the pathogens to thrive and enhance their virulence. Therefore, a therapeutic approach that can inhibit the QS of *P. aeruginosa* would be effective in suppressing *P. aeruginosa* biofilm formation and reducing the production of its virulence factors, resulting in lower and less frequent exacerbation in bronchiectasis patients [16].

Quercetin (QUE)—a natural flavonoid known for its vast therapeutic activities (e.g., antioxidant, antidiabetic, antimicrobial, anticancer) and widely found in fruits and vegetables—has been demonstrated recently as an effective inhibitor of QS in *P. aeruginosa*. The QS inhibition in the presence of QUE was manifested in reduced biofilm formation, lower production of its virulence factors (e.g., pyocyanin, protease, elastase), and reduced swimming motility [17,18,19]. Significantly, a combinatorial therapy of QUE and antibiotics has been demonstrated in vitro to exhibit synergistic killing effects against antibiotic-resistant *P. aeruginosa* strains [20]. Hence, the incorporation of QUE into bronchiectasis treatment has the potential to reduce the treatment’s current overreliance on antibiotics [21].

Inhaled delivery route of QUE is recommended for this purpose as it enables targeted delivery of QUE to the infected lung sites. Another compelling reason for inhaled QUE lies in the fact that oral administration of QUE is notoriously known for its resultant low bioavailability due to the significant first-pass metabolism of QUE in the gut and liver [22]. Successful clinical application of inhaled QUE, nevertheless, remains challenging due to its low solubility in physiological fluid (<100 µg/mL) [23], which would severely limit the amount of QUE exposure to the biofilm cells. Numerous solubility enhancement strategies for QUE have been developed, for example, via nanonization [24,25], cocrystallization [26], amorphous solid dispersion [25,27], cyclodextrin inclusion complex [25], and chemical conjugation [28]. However, a majority of these strategies are either energy and time-intensive (e.g., nanonization and amorphization) or involve multiple synthesis steps and heavy use of organic solvents (e.g., chemical conjugation).

Herein we proposed a simple, rapid, energy-minimal, and solvent-free solubility enhancement strategy of QUE in the form of an amorphous QUE-chitosan nanoparticle complex (or nanoplex in short). The nanoplex was prepared by a simple drug-polyelectrolyte complexation technique that involved only bulk mixing of the ionized drug solution (i.e., QUE) and the oppositely charged polyelectrolytes (PE) (i.e., chitosan, CHI). CHI was selected as the PE for two reasons; first, CHI is a strong polycation hence capable of electrostatic binding with the anionic QUE, and second, CHI is known to possess potent antibacterial activities making it an ideal adjuvant for incorporation into bronchiectasis’ treatment regimen [29,30].

**Figure 1:**
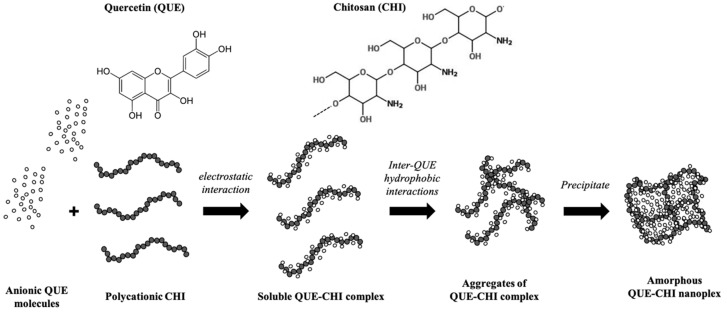
Schematics of the quercetin (QUE)– chitosan (CHI) nanoplex formation.

As illustrated in Figure 1, the QUE–CHI nanoplex formation began with the formation of soluble QUE–CHI complexes upon mixing of the QUE and CHI solutions as a result of the QUE–CHI electrostatic interactions. Aggregates of the soluble QUE–CHI complex were then formed due to inter-drug hydrophobic interactions among the bound QUE molecules. The complex aggregates later precipitated out of the solution to form amorphous QUE–CHI nanoplex upon reaching a critical mass. The amorphous form of the nanoplex was attributed to the restricted molecular mobility of the QUE molecules bound to the CHI chains, which in turn prevented them from re-arranging to the ordered crystalline structure upon precipitation [31]. Owing to its metastable amorphous form and nanoscale size, the nanoplex was capable of generating a highly supersaturated concentration of the drug upon dissolution, resulting in a high drug’s kinetic solubility that was multifold higher than the thermodynamic solubility of its crystalline counterpart [32].

The objectives of the present work were as follows: First, we aimed to determine the optimal preparation pH for the QUE–CHI nanoplex as characterized by the resultant (i) complexation efficiency of QUE, (ii) QUE payload, (iii) production yield, and (iv) size and zeta potential. Second, using the QUE–CHI nanoplex prepared at the optimal pH, we aimed to examine the impacts of complexation with CHI on the QUE’s (i) dissolution rate, (ii) solubility enhancement, (iii) antibacterial activity towards *P. aeruginosa*, and (iv) in vitro cytotoxicity towards human lung cancer cells. Third, we aimed to evaluate the *P. aeruginosa* QS inhibition capacity of the optimal QUE–CHI nanoplex in comparison to that of the native QUE. The QS inhibition capacity of the nanoplex was characterized by the resultant (i) swimming motility, (ii) rate of biofilm formation, and (iii) production of *P. aeruginosa* virulence factor (i.e., pyocyanin).

## 2. Results and Discussion

### 2.1. Optimal Preparation pH

#### 2.1.1. Effects of pH on Size and Zeta Potential

**Figure 2:**
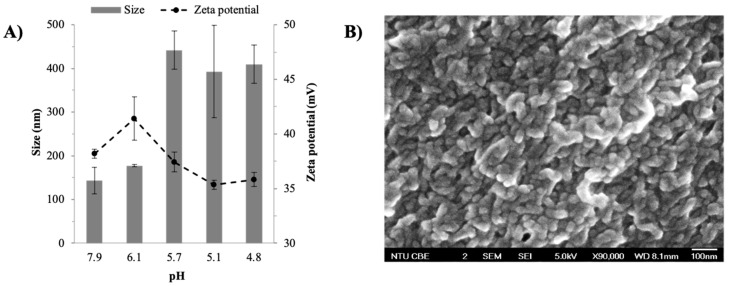
(**A**) Effect of pH on size, zeta potential as measured by dynamic light scattering microscopy (DLS); (**B**) FESEM image of the QUE–CHI nanoplex.

The effects of pH of the mixed QUE and CHI solutions were investigated between pH 4.8 and 7.9 as QUE was only deprotonated at pH above 4.0 [33] while it was prone to chemical oxidation at pH > 9.5 [34]. The DLS results showed that the QUE–CHI complexation led to the formation of particulate complexes with sizes in the nanometer range (150–400 nm) and zeta potentials of 36–41 mV (Figure 2A). The positive zeta potential indicated the presence of cationic CHI on the nanoplex surface. The QUE–CHI nanoplex size was found to be larger (≈400 nm) at pH ≤ 5.7 compared to ≈150 nm at pH 6.1 and 7.9, whereas the zeta potential was minimally affected by the pH variation. On this note, the control runs in which the QUE solution (0.25% *w*/*v*) was mixed with AA solution without CHI did not produce any precipitates, thus the nanoparticles produced was resulted from the QUE–CHI complexation. The same result of no precipitation was also observed for the control run in which the CHI solution (0.296% *w*/*v*) was mixed with the KOH solution without QUE.

The nanoscale size of the QUE–CHI complex was verified by the FESEM analysis that showed the appearance of nanoparticles with individual sizes smaller than 100 nm (Figure 2B). The QUE–CHI nanoplex prepared at pH 6.1 was used as the representative sample for FESEM. In comparison to the FESEM, the larger size obtained by DLS was likely caused by the agglomeration tendency of the nanoplex in its aqueous suspension form. The QUE–CHI nanoplex was revealed from the FESEM image to possess an elongated shape. It is worth mentioning that the particles observed in the FESEM could not plausibly represent a mixture of QUE and CHI precipitates because the control experiments had shown that neither QUE nor CHI precipitated in the absence of the other in the range of pH and concentrations of QUE and CHI investigated here. Thereby, the precipitation only occurred upon QUE–CHI complexation.

#### 2.1.2. Effects of pH on the Preparation Efficiency and QUE Payload

**Figure 3:**
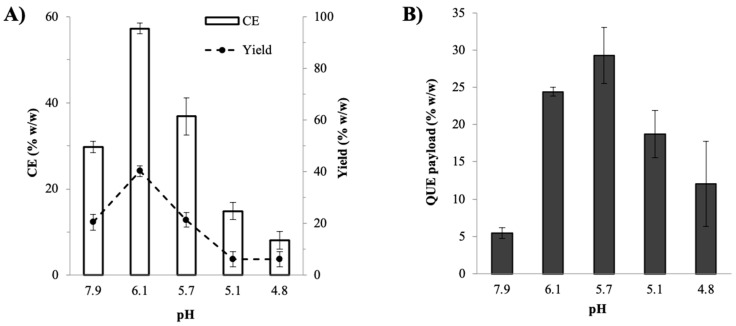
Effect of pH on (**A**) complexation efficiency (CE), yield and (**B**) QUE payload of the QUE–CHI nanoplex.

The effects of pH on the preparation efficiency of the QUE–CHI nanoplex were examined from the resultant complexation efficiency (CE) of QUE, production yield, and QUE payload. The results in Figure 3A show that the CE gradually increased with increasing pH from 8.1 ± 2.0% at pH 4.8 to 36.9 ± 4.3% (*w*/*w*) at pH 5.7. The CE reached the maximum at pH 6.1 with CE equal to 57.3 ± 1.3%. The CE, however, decreased to 29.7 ± 1.3% upon a further increase in pH to 7.9. The higher CE observed with increasing pH was attributed to the higher degree of ionization of QUE at higher pH owed to increased dissociations of its hydroxyl groups [35]. Whereas the sharp decrease in the CE at pH 7.9 was caused by the lower degree of ionization of CHI, which is a basic PE, at non-acidic pH above its pKa, resulting in fewer CHI charges available for complexation with the ionized QUE molecules. This led to fewer QUE molecules were transformed into the QUE–CHI complex.

A similar trend as a function of pH was observed in the production yield, where the yield gradually increased from 6.2 ± 2.9% (*w*/*w*) at pH 4.8 to reach the maximum at 40.2 ± 2.1% at pH 6.1, after which the yield decreased to 20.5 ± 3.0% at pH 7.9 (Figure 3A). The results on yields suggested that the yield-pH relationship was dictated by the CE of QUE. The QUE payload was also shown to exhibit a similar relationship with the pH, where the payload gradually increased from 12.1 ± 5.7% (*w*/*w*) at pH 4.8 to a peak value of 29.3 ± 3.7% at pH 5.7 before it decreased to 5.4 ± 0.7% at pH 7.9 (Figure 3B). The increase in the QUE payload with increasing pH was not unexpected as the lower CE observed at lower pH translated to a smaller presence of QUE in the QUE–CHI complex, where the charged binding sites of CHI were mostly unoccupied by the QUE molecules.

Based on the results of the CE, yield, and QUE payload, the optimal pH for the QUE–CHI nanoplex preparation was determined at pH 6.1, at which the nanoplex exhibited size and zeta potential of 177 ± 2 nm and 41 ± 2 mV, respectively, with PDI of 0.241 ± 0.020 indicating its largely uniform size. At the optimal pH, approximately 57% of QUE was successfully transformed into the nanoplex at ≈40% overall yield, resulting in the QUE payload of 24.4 ± 0.6% (*w*/*w*) with the rests of the nanoplex mass was made up of CHI.

### 2.2. Physical Characteristics of the Optimal QUE–CHI Nanoplex

#### 2.2.1. FTIR

**Figure 4:**
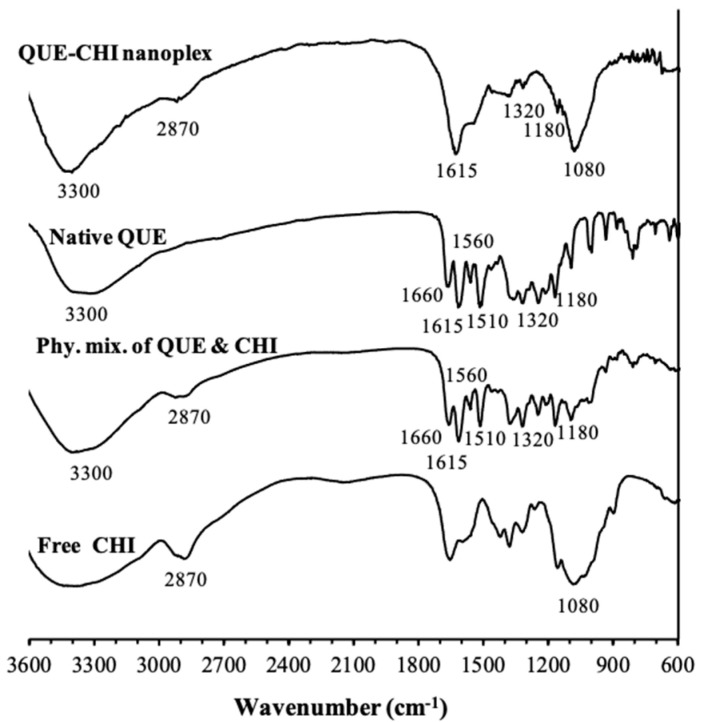
FTIR spectra of the QUE–CHI nanoplex and its constituents.

The presence of QUE in the QUE–CHI nanoplex prepared at the optimal pH was verified in the FTIR analysis by the appearance of the characteristic peaks of QUE at 1320 and 1615 cm^−1^ in the FTIR spectrum of the nanoplex (Figure 4). These two peaks were attributed, respectively, to the OH bending of the phenolic group and C=C stretching of the aromatic rings, both of which were unique to QUE and not present in CHI [36]. The presence of QUE was also evident by the presence of a strong, broad peak centered at around 3300 cm^−1^ attributed to OH stretching of the five hydroxyl groups of QUE. These three peaks expectedly also appeared in the FTIR spectrum of the native QUE. The presence of CHI in the QUE–CHI nanoplex was verified by the appearance of peaks at 1080 and 2870 cm^−1^ attributed to the C-N stretching and N-H stretching of the amine groups of CHI, respectively. The amine peaks were also observed in the FTIR spectrum of the free CHI, as expected.

The complexation between QUE and CHI in the nanoplex could be discerned by the almost disappearance of the peak at 1180 cm^−1^ attributed to the C-O stretching of the phenols of QUE in the FTIR spectrum of the QUE–CHI nanoplex. The same peak at 1180 cm^−1^ was clearly visible in the FTIR spectra of the native QUE and the physical mixture of QUE and CHI in which complexation did not take place. We attributed the reduced intensity of the peak at 1180 cm^−1^ in the nanoplex spectrum to the electrostatic interaction between the hydroxyl groups of the phenols in QUE and the amine groups of CHI. A similar reduction in the intensity, though to a lesser extent, was also observed for the OH bending peak of the phenols of the QUE at 1320 cm^−1^. Comparing the FTIR spectrum of the QUE–CHI nanoplex with that of the physical mixture of QUE and CHI revealed further differences between the two suggesting that QUE–CHI interactions via electrostatic or hydrogen bond interactions indeed took place. Specifically, the physical mixture’s spectrum (as well as the native QUE’s) exhibited peaks at 1660 cm^−1^ attributed to the C=O stretching of the aryl ketonic group of QUE and at 1560 and 1510 cm^−1^ attributed to C=C stretching of the aromatic rings of QUE, both of which did not appear in the QUE–CHI nanoplex spectrum.

#### 2.2.2. PXRD

**Figure 5:**
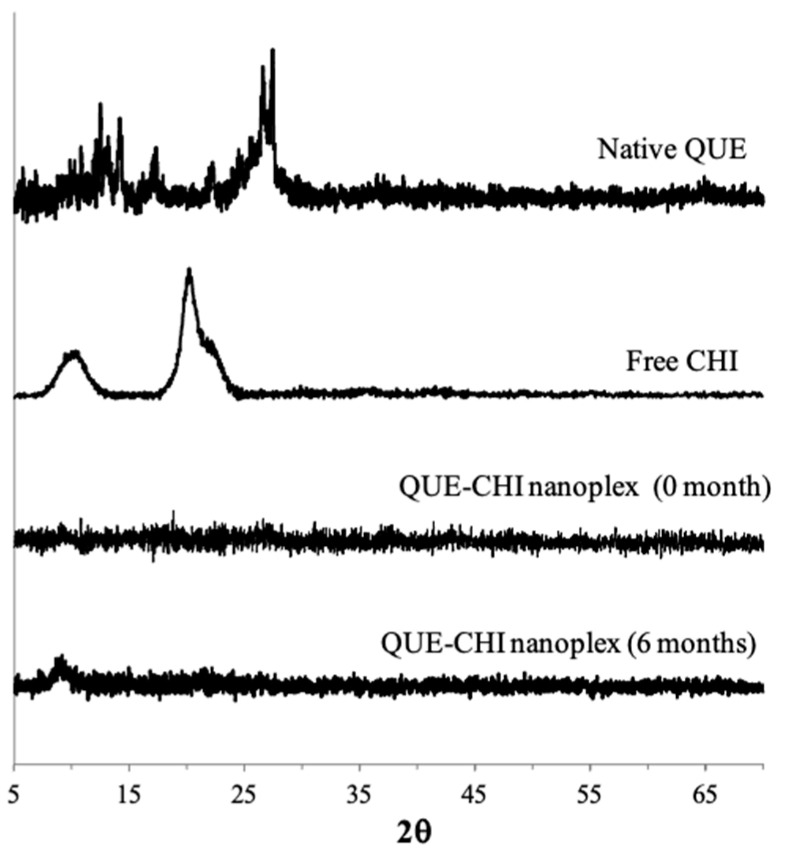
Powder X-ray diffraction (PXRD) patterns of the QUE–CHI nanoplex before and after storage.

The PXRD analysis in Figure 5 revealed that the native QUE used in this study and the QUE–CHI nanoplex preferentially existed as amorphous solids. In contrast to the native QUE, whose PXRD pattern exhibited a few strong-intensity peaks characteristic of crystalline contents at 2θ≈15 and 28, strong-intensity peaks were completely absent in the PXRD pattern of the QUE–CHI nanoplex, hence indicating its fully amorphous contents. A fully amorphous nanoplex was not unexpected as CHI, which made up roughly 75% of the nanoplex mass, inherently existed as amorphous solids as indicated by the broad, amorphous halos in the PXRD pattern of the free CHI. Importantly, the electrostatic stabilization provided by CHI was sufficient to keep the amorphous QUE in the nanoplex stable during the prolonged storage of six months, where no recrystallization events were observed from the PXRD patterns after storage.

### 2.3. Dissolution Characteristics

**Figure 6:**
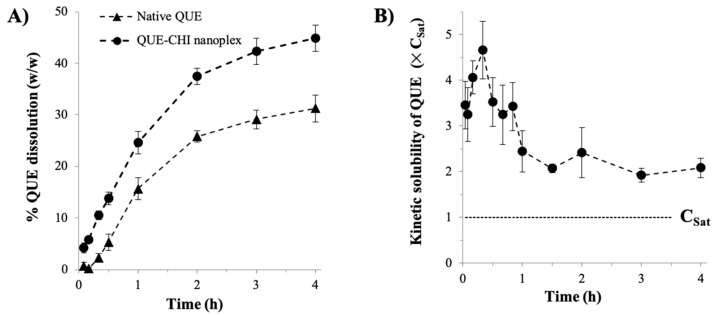
(**A**) dissolution of the QUE–CHI nanoplex versus the native QUE; (**B**) QUE kinetic solubility.

The QUE dissolution from the QUE–CHI nanoplex under sink condition was found to be faster compared to the native QUE dissolution with 24.6 ± 2.2% and 44.9 ± 2.5% (*w*/*w*) dissolutions after 1 h and 4 h, respectively, compared to 15.6 ± 2.1% and 31.2 ± 2.6% for the native QUE after the same periods (Figure 6A). The faster dissolution rate of the QUE–CHI nanoplex was attributed to its nanoscale size that increased the specific surface areas available for dissolution. The higher amorphous contents of the nanoplex compared to the native QUE also contributed to the nanoplex’s faster dissolution rate as dissolution from amorphous solids did not have to overcome the crystal lattice energy. The faster dissolution rate enabled the QUE–CHI nanoplex to produce a peak kinetic solubility after 20 min that was (4.7 ± 0.6)× higher than C_Sat_ of the native QUE (Figure 6B). The peak kinetic solubility, however, was short-lived as the kinetic solubility immediately decreased to (3.5 ± 0.5) × C_Sat_ 10 min after reaching the peak due to precipitation of the supersaturated QUE solution. While the kinetic solubility continued to decrease with time, it remained at least twice higher than C_Sat_ over 4 h. In this regard, polymeric crystallization inhibitors, such as hydroxypropylmethylcellulose, could be incorporated into the nanoplex dosage formulation to prolong the kinetic solubility of QUE [37].

### 2.4. Antibacterial Activity

The MIC of the QUE–CHI nanoplex against the planktonic *P. aeruginosa* PAO1 was determined to be equal to 500 µg/mL with OD_600_ = 0.048 ± 0.067, which was found to be similar to the MIC of the native QUE with OD_600_ = 0.097 ± 0.029 (Table 1). The present MIC results were in agreement with the previously reported MIC of QUE against *P. aeruginosa* PAO1 (>256 µg/mL) [17]. The comparable MIC values between the native QUE and the QUE–CHI nanoplex signified that the antibacterial activity of QUE was not adversely affected by its complexation with CHI, which boded well for the potential applications of the QUE–CHI nanoplex in infection therapy. It was worth noting that the intended role of QUE here was not as an antibacterial agent that eradicated bacteria but rather as a QS-inhibiting agent that in turn suppressed the bacterial biofilm formation without necessarily killing the bacteria. Therefore, it was not mandatory to have QUE exposure above the MIC to achieve the desired QS inhibition effects, as we showed in the next section.

In the range of QUE concentration investigated (i.e., 100–500 µg/mL), the QUE–CHI nanoplex contained approximately 300–1500 µg/mL of CHI, which was calculated based on roughly 25% (*w*/*w*) QUE payload in the nanoplex. As CHI was well known for its antibacterial activity [38], the presence of CHI in the nanoplex may contribute to the QUE–CHI nanoplex’s antibacterial activity. In this regard, the MIC of the free CHI against *P. aeruginosa* was determined to be equal to 125 µg/mL (Table A1 in Appendix A). Hence it was lower than the lowest CHI concentration present in the QUE–CHI nanoplex (i.e., 300 µg/mL). The results of the QUE–CHI nanoplex’s antibacterial activity, however, showed that the presence of CHI above its MIC did not necessarily lead to higher antibacterial activity for the nanoplex. Specifically, OD_600_ values of the cell suspensions treated with the QUE–CHI nanoplex and the native QUE were found to be highly similar, denoting comparable cell growths in the two treatments. Thus, the presence of CHI in the nanoplex was not found to enhance its antibacterial activity, suggesting that the complexation with QUE had an adverse effect on the antibacterial activity of CHI, albeit a more thorough study was needed to reaffirm this.

### 2.5. Anticancer Activity

**Figure 7:**
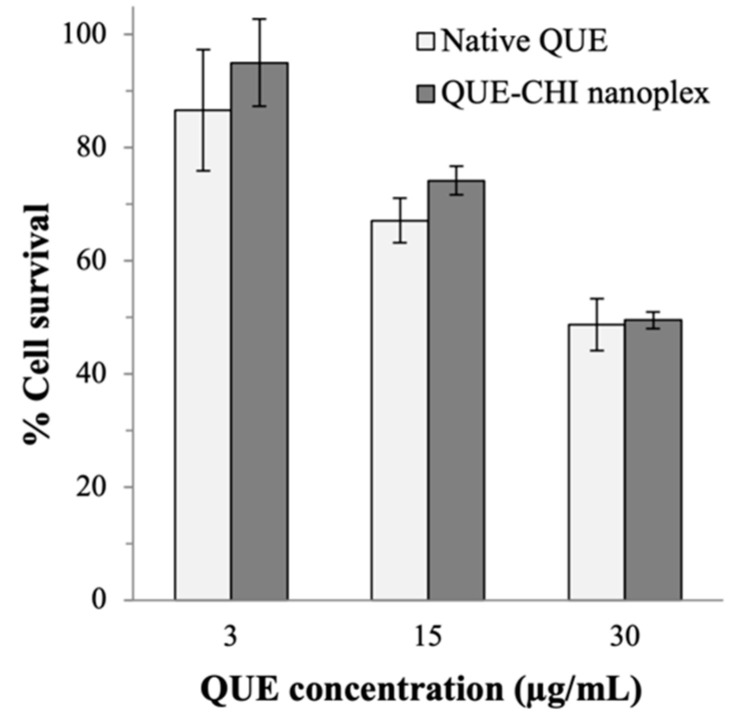
Cytotoxicity of the QUE–CHI nanoplex and native QUE towards A549 lung cancer cells.

The QUE–CHI nanoplex and the native QUE exhibited similar cytotoxicity profiles towards the A549 human lung cancer cells in the range of the QUE concentrations investigated (i.e., 3–30 µg/mL) (Figure 7). Specifically, at QUE = 3 µg/mL, both the native QUE and the QUE–CHI nanoplex exhibited minimal cytotoxicity towards the A549 cells with cell survivals of 87 ± 10% and 95 ± 7%, respectively. At a higher QUE exposure of 10 µg/mL, the cell survivals decreased to 67 ± 4% and 74 ± 2% for the native QUE and the QUE–CHI nanoplex, respectively. The cell survivals decreased further to 49 ± 4% and 50 ± 1% for the native QUE and the QUE–CHI nanoplex, respectively, at QUE exposure of 30 µg/mL. In this regard, to the best of our knowledge, the amount of QUE recovered in the lung after inhaled QUE delivery has not been experimentally investigated before. Therefore, we carried out the cytotoxicity test in the QUE concentration range that was roughly between two to twenty times higher than the QUE concentration recovered in the lung after oral QUE delivery (i.e., ≈1.5 µg/mL) [39], as we anticipated that the inhaled delivery route would lead to a higher QUE concentration in the lung owed to avoidance of the first-pass metabolism.

**Figure A1:**
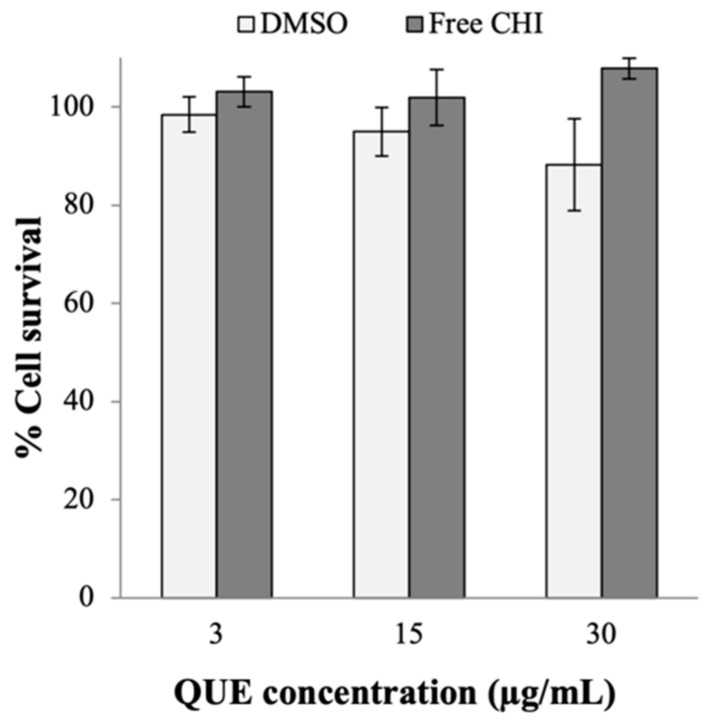
Cytotoxicity of the free CHI and dimethyl sulfoxide (DMSO) towards the A549 cells.

To investigate whether CHI in the nanoplex or DMSO used to prepare the native QUE solution contributed to the cytotoxicity results, the cytotoxicity of the free CHI and pure DMSO were examined. The results in Figure A1 in Appendix A showed that both CHI and DMSO had negligible cytotoxicity towards the A549 cells. Therefore, QUE was responsible for the cytotoxicity of the lung cancer cells exhibited by the QUE–CHI nanoplex. Significantly, the similar cytotoxicity between the native QUE and the nanoplex indicated that the QUE complexation with CHI had a negligible impact on the anticancer activity of QUE.

### 2.6. QS Inhibition

#### 2.6.1. Swimming Motility

**Figure 8:**
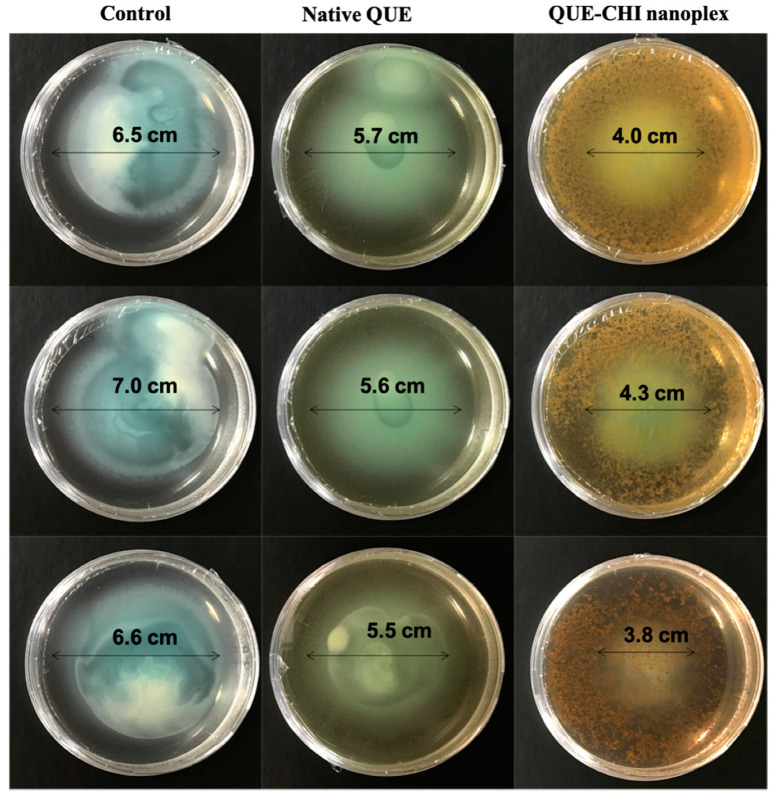
Swimming motility of *P. aeruginosa* in the presence of QUE (note: top, middle, and bottom panels represent the results from three independent replicates).

QS-regulated swimming motility was essential in the first stage of bacterial invasion and growth for biofilm formation in *P. aeruginosa*. Therefore, the QS inhibition afforded by the QUE–CHI nanoplex was first evaluated by the bacterial swimming motility in the presence of the nanoplex. The results in Figure 8 from three independent replicates showed that the bacteria in the plate in which QUE was absent (i.e., Control) exhibited a swimming zone diameter of 6.7 ± 0.3 cm. The presence of QUE at 200 µg/mL either in the form of the native drug or the nanoplex reduced the swimming motility of the bacteria as reflected by the shorter swimming zone diameters of 5.6 ± 0.1 cm and 4.0 ± 0.3 cm for the plates containing the native QUE and the QUE–CHI nanoplex, respectively. This translated to approximately 17% and 41% reductions in the swimming zone diameters in the presence of the native QUE and the QUE–CHI nanoplex, respectively. On this note, the dark yellowish color in the outer ring of the plate containing the QUE–CHI nanoplex was due to the color of the nanoplex dispersed in the agar.

The superior suppression of the swimming motility exhibited by the QUE–CHI nanoplex was attributed to its higher solubility compared to the native drug, resulting in a higher QUE exposure in the plate. In addition to the higher solubility, the superior suppression of the swimming motility by the QUE–CHI nanoplex could also be attributed to the CHI fraction of the nanoplex. In this regard, the QS-inhibiting activity of CHI in *P. aeruginosa* has been recently established in a number of studies [40,41]. Moreover, besides its own QS-inhibiting activity, the inclusion of CHI also has been reported to enhance the QS-inhibiting activity of the flavonoid group of chemicals, which QUE belonged to [42].

#### 2.6.2. Rate of Biofilm Formation

**Figure 9:**
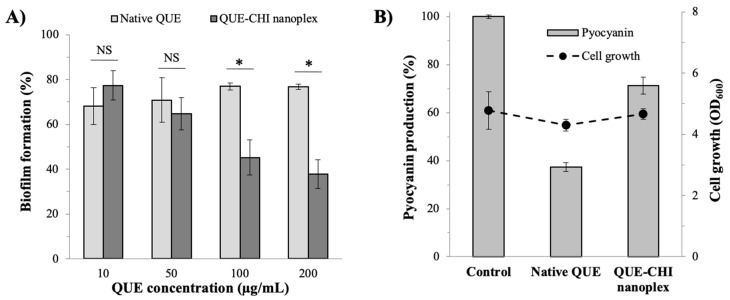
(**A**) Rate of biofilm formation and (**B**) pyocyanin production in the presence of QUE (NS = not statistically significant, * *p* ≤ 0.05).

The rate of *P. aeruginosa* biofilm formation in the presence of QUE, which was reported as the percentage relative to the biofilm formation of the control run (i.e., no QUE), is shown in Figure 9A. For both the native QUE and the QUE–CHI nanoplex, the effect of the QUE concentration on the biofilm formation was investigated between 10 and 200 µg/mL, which were well below its MIC value (i.e., 500 µg/mL), denoting very minimal eradication of the bacterial cells.

In the presence of the native QUE, the rates of biofilm formation were equal to 68 ± 8% and 71 ± 9% at QUE exposures of 10 and 50 µg/mL, respectively, denoting successful inhibition of the biofilm formation. The biofilm formation rate increased to about 77% at QUE exposures of 100 and 200 µg/mL, indicating that the inhibition of the biofilm formation did not scale proportionally to the QUE exposure. These results were in agreement with the results of Ouyang et al. [17], who reported that the highest QUE exposure did not necessarily lead to the highest inhibition of the biofilm formation.

In the presence of the QUE–CHI nanoplex, the rates of biofilm formation at QUE exposures of 10 and 50 µg/mL were equal to 77 ± 6% and 65 ± 7%, respectively. These values were comparable to the biofilm formation rates observed in the presence of the native QUE at the same QUE concentrations. In fact, the difference in the rates of biofilm formation between the native QUE and the QUE–CHI nanoplex exposures at 10 and 50 µg/mL was statistically insignificant.

In contrast, at QUE exposures of 100 and 200 µg/mL, the rates of biofilm formation in the presence of the QUE–CHI nanoplex were significantly lower at 45 ± 8% and 38 ± 6%, respectively, signifying the superiority of the QUE–CHI nanoplex to the native QUE in inhibiting the biofilm formation. The superior biofilm formation inhibition exhibited by the nanoplex was attributed to the better suppression of the swimming motility in its presence and other QS inhibitions afforded by both QUE and CHI. On this note, unlike the trend with the native QUE, the inhibition of the biofilm formation in the presence of the QUE–CHI nanoplex was found to be proportionally dependent on the concentration of the QUE–CHI nanoplex used. As increasing the QUE concentration on its own was not found to necessarily lead to less biofilm formation, the better suppression of the biofilm formation at higher QUE–CHI nanoplex concentrations ought to be attributed to the increased QS-inhibition contribution from CHI.

#### 2.6.3. Virulence Factor Production

The QS inhibition afforded by the QUE–CHI nanoplex was examined further by the production of one of the virulence factors of *P. aeruginosa*, i.e., pyocyanin—a phenazine notoriously known to be the virulence factor responsible for causing the hosts’ cells and tissues damage and persistent infection [43]. The results in Figure 9B shows that the pyocyanin production in the presence of the native QUE at 200 µg/mL was diminished at only 37.3 ± 1.9% compared to the pyocyanin production in the control run (i.e., 100%). In the presence of the QUE–CHI nanoplex also at 200 µg/mL, the pyocyanin production was higher at 71.3 ± 3.5%, hence indicating that the QUE–CHI nanoplex was inferior to the native QUE in suppressing the pyocyanin production. As the cell growth among the three runs was similar (OD_600_ ≈4.3–4.7) (Figure 9B), the variations in the pyocyanin production between the two treatments were confirmed to be caused by the QS inhibition and not due to lower production because of cell deaths.

## 3. Materials and Methods

### 3.1. Materials

*Materials for QUE–CHI nanoplex preparation and characterization*: anhydrous quercetin (QUE) (purity ≥ 95%), chitosan (CHI) (MW 50–190 kDa), glacial acetic acid (AA), potassium hydroxide (KOH), acetonitrile, formic acid, cellulose dialysis bag with MW cutoff of 14 kDa, dimethyl sulfoxide (DMSO), chloroform were purchased from Sigma-Aldrich (Singapore). *Materials for bacterial cell culture*: *P. aeruginosa* PAO1 strain was purchased from ATCC (USA). Mueller–Hinton broth (MHB), tryptone, and phosphate-buffered saline (PBS, pH 7.4) were purchased from BD Diagnostics (Singapore), Gibco (USA), and Sigma-Aldrich (Singapore), respectively. *Materials for cytotoxicity tests*: A549 adenocarcinomic human lung epithelial cells were purchased from ATCC (USA). Penicillin–streptomycin and CellTiter-Blue were purchased from PAA Laboratories (Austria) and Promega (Singapore), respectively. Dulbecco’s modified Eagle’s medium (DMEM), fetal bovine serum, Luria–Bertani (LB) broth and agar were purchased from GE Healthcare Life Sciences (USA).

### 3.2. Methods

#### 3.2.1. Preparation of QUE–CHI Nanoplex

CHI having pKa of 6.5 [44] was dissolved at 2.96 mg/mL in aqueous AA solution to form polycations with a charge density of 5.58 × 10^−6^ mol-charge/mg attributed to protonation of its amine group. QUE having multiple pKa of 5.7, 7.1, 8.0, 9.9, and 11.0 [33] was dissolved at 2.5 mg/mL in 0.1M KOH under 1 min vortexing to deprotonate the hydroxyl groups of QUE, resulting in the formation of anionic QUE molecules. The QUE solution was immediately mixed with an equal volume of the CHI solution under gentle stirring to minimize the alkaline degradation of QUE that could lead to its oxidation [45]. The optimal pH was determined by varying the AA concentration of the CHI solution between 0.4 and 1.0% (*v*/*v*) to produce pH of the mixed solutions between 4.8 and 7.9. In this pH range, only two hydroxyl groups of QUE were deprotonated [33], resulting in QUE’s charge density of 6.62 × 10^−6^ mol-charge/mg. Control experiments in which (1) the QUE solution was mixed with the AA solution without CHI and (2) the CHI solution was mixed with the KOH solution without QUE were performed. Next, the resultant QUE–CHI nanoplex solution underwent two cycles of centrifugation (14,000× *g* for 10 min) and washing with deionized (DI) water to remove excess QUE and CHI that did not form the nanoplex. The washed nanoplex was redispersed in DI water for characterizations. The dry-powder form of the nanoplex was produced by freeze-drying for 24 h at −52 °C and 0.05 mbar in Alpha 1–2 LD Plus freeze-dryer (Martin Christ, Germany).

#### 3.2.2. Physical Characterizations of QUE–CHI Nanoplex

The size, polydispersity index (PDI), and zeta potential of the QUE–CHI nanoplex were characterized in triplicates by dynamic light scattering microscopy (DLS) after 100 × dilutions using Brookhaven 90 Plus nanoparticle size analyzer (Brookhaven Instruments Corporation, NY, USA). The nanoplex size was verified by field emission scanning electron microscope (FESEM) (JSM-6700 F, JEOL, Tokyo, Japan) using air-dried nanoplex suspension as the sample. The QUE payload defined as the mass of QUE per unit mass of the nanoplex was determined from four replicates by dissolving a known mass of the dry-powder nanoplex in acetonitrile, after which the amount of QUE in the solution was determined by high-performance liquid chromatography (HPLC) using Agilent 1100 (Agilent Technologies, CA, USA). The HPLC analysis was performed in the ZORBAX Eclipse Plus C18 column (250 × 4.6 mm, 5 µm particle size) at 294 nm. Acetonitrile: 0.1% formic acid (85:15 *v*/*v*) solution was used as the mobile phase at 0.8 mL/min, resulting in the retention time of QUE at approximately 2.8 min.

The complexation efficiency (CE) defined in Equation (1) was characterized in triplicates by determining the mass of QUE that was transformed to the nanoplex from the difference between the initial amount of QUE added and the amount of QUE remaining in the supernatant after the first centrifugation. The amount of QUE in the supernatant was determined by HPLC as previously described after 20× dilution of the supernatant in acetonitrile. The production yield defined in Equation (2) was determined in triplicates by taking the ratio of the dry-powder mass of the QUE–CHI nanoplex produced to the initial masses of QUE and CHI in the feed.

(1) $$\mathrm{CE}\;\left(\%\;w/w\right)=\;\frac{\mathrm{Mass}\;\mathrm{of}\;\mathrm{QUE}\;\mathrm{that}\;\mathrm{was}\;\mathrm{transformed}\;\mathrm{to}\;\mathrm{the}\;\mathrm{QUE}-\mathrm{CHI}\;\mathrm{nanoplex}\;}{\mathrm{Initial}\;\mathrm{mass}\;\mathrm{of}\;\mathrm{QUE}\;\mathrm{added}}\;\times \;100$$

(2) $$\mathrm{Yield}\;\left(\%\;w/w\right)=\;\frac{\mathrm{Mass}\;\mathrm{of}\;\mathrm{QUE}-\mathrm{CHI}\;\mathrm{nanoplex}\;\mathrm{produced}}{\mathrm{Initial}\;\mathrm{masses}\;\mathrm{of}\;\mathrm{QUE}\;\mathrm{and}\;\mathrm{CHI}\;\mathrm{added}}\;\times \;100$$

The presence of QUE in the nanoplex and its interaction with CHI was examined by Fourier-transform infrared spectroscopy (FTIR) from 450 and 4000 cm^−1^ at 4 cm^−1^ spectral resolution in Spectrum One (PerkinElmer, MA, USA). The FTIR analysis was performed for the native QUE, free CHI, QUE–CHI nanoplex, and physical mixture of the QUE and CHI (1:3 by mass). The amorphous form of the QUE–CHI nanoplex were examined by powder X-ray diffraction (PXRD) immediately after its preparation and after six-month storage at 25 °C and 60% relative humidity. The PXRD analysis was performed using D8 Advance X-ray diffractometer equipped with Cu Kα radiation (Bruker, Ettlingen, Germany) from 5° to 70° (2θ) with a step size of 0.02°/s. The PXRD analysis was also performed for the native QUE.

#### 3.2.3. Kinetic Solubility and Dissolution Rate

Thermodynamic saturation solubility (C_Sat_) of the native QUE in PBS was determined in triplicates by adding excess native QUE (20 mg) to 20 mL PBS in an opaque bottle placed in a shaking incubator at 37 °C. After 24 h incubation, 5 mL aliquot was withdrawn and subsequently centrifuged (14,000× *g* for 5 min) and syringe filtered (0.2 µm pore size) to remove the undissolved QUE. The QUE concentration in the filtered solution was quantified by HPLC, from which C_Sat_ of approximately 145 ± 10 µg/mL was determined.

The kinetic solubility of the amorphous QUE–CHI nanoplex as a function of time was determined by adding the QUE–CHI nanoplex suspension in excess at 10 × C_Sat_ to 20 mL PBS in an opaque bottle placed in a shaking incubator at 37 °C. At specific time points over 4 h, 800 µL aliquot was withdrawn and syringe filtered (0.2 µm pore size), followed by 50 × dilutions with fresh PBS to prevent QUE precipitation from the supersaturated solution. The QUE concentration in the diluted aliquot was then quantified by HPLC. The kinetic solubility was reported as the ratio of the supersaturated QUE concentration generated by the QUE–CHI nanoplex to C_Sat_ of the native QUE.

The QUE dissolution from the QUE–CHI nanoplex was characterized under a sink condition (i.e., 1/5 of C_Sat_) by adding the QUE–CHI nanoplex suspension containing roughly 1.5 mg of QUE into a dialysis bag immersed in 50 mL PBS in an opaque bottle placed in a shaking incubator at 37 °C. At specific time points over 4 h, 1 mL aliquot was withdrawn, and the same volume of fresh PBS was added back to the dissolution bottle. The aliquot was centrifuged and syringe filtered, after which the QUE concentration in the filtered solution was determined by HPLC. The dissolution experiment was repeated for the native QUE for comparison. The kinetic solubility and dissolution were characterized using three independent batches of the optimal QUE–CHI nanoplex.

#### 3.2.4. Antibacterial Activity

Antibacterial activity of the QUE–CHI nanoplex against *P. aeruginosa* PAO1 was characterized by its minimum inhibitory concentration (MIC). The MIC was performed in triplicates by the microbroth dilution method. Briefly, an overnight inoculum of *P. aeruginosa* PAO1 was adjusted to 0.5 McFarland standards and diluted in MHB for 1.0 × 10^6^ CFU/mL. Next, 200 μL of the cell suspension was added to a 96-well microplate, after which 200 μL of the QUE–CHI nanoplex suspension was added to the wells. The range of QUE concentration investigated was from 50 to 500 µg/mL. The cells were then incubated at 37 °C for 24 h. The bacterial cell growth was characterized from the optical density at 600 nm (OD_600_) using a microplate reader (Synergy HT, Biotek, VT, USA), where OD_600_ < 0.1 signified no visible cell growth. The MIC of the native QUE dissolved in 5% (*v*/*v*) DMSO was determined using the same protocols.

#### 3.2.5. Cytotoxicity towards the Human Lung Cancer Cells

The A549 cells were cultivated in a 96-well tissue culture microplate at 10^5^ cells/mL using DMEM supplemented with 10% (*w*/*v*) fetal bovine serum and 1% (*w*/*v*) penicillin–streptomycin solution as the culture medium. The cells were incubated in a 5% CO_2_ incubator for 24 h at 37 °C. Afterward, the QUE–CHI nanoplex suspension containing QUE concentration in the range of 10–100 µM was added to the cells. The cells exposed to the nanoplex were then incubated at 37 °C for 24 h. After 24 h, 20 µL CellTiter-Blue^®^ reagent was added to the cells, followed by further incubation for 4 h. The viable cells were quantified from the concentration of resorufin determined by UV-vis spectrophotometry at 570 nm. The cytotoxicity of the nanoplex was reported based on three replicates in terms of the cell survival percentage, which was calculated from the ratio of OD_570_ of the exposed cells to OD_570_ of the unexposed cells (i.e., placebo). In addition, the cytotoxicity of (i) the native QUE dissolved in 0.15% (*v*/*v*) DMSO, (ii) free CHI, and (iii) pure DMSO were determined for comparison.

#### 3.2.6. Rate of Biofilm Formation

The rate of biofilm formation was characterized in triplicates following the crystal violet staining methods presented in Ouyang et al. [17]. Briefly, the *P. aeruginosa* PAO1 cells were inoculated in LB broth overnight, after which they were diluted to OD_600_ of 0.05. The resultant planktonic cell suspension was then incubated in a 24-well microplate in the presence of the QUE–CHI nanoplex containing QUE concentrations in the range of 10 to 200 µg/mL. A control run in which the cells were incubated in the absence of the nanoplex was carried out as the control run. After 24 h incubation at 37 °C, the biofilm cells formed in the microplate were washed thrice with PBS to remove the non-adherent cells. Next, the biofilm cells were stained with 0.1% (*w*/*v*) crystal violet solution for 15 min. The cells were then washed thrice with PBS to remove the excess stain, followed by 15 min air drying. The crystal violet bound to the biofilm cells was extracted with 30% (*v*/*v*) AA solution, after which its amount was quantified using a microplate reader at OD_590_. The biofilm formation in the presence of the QUE–CHI nanoplex was characterized by the ratio of OD_590_ of the exposed cells to OD_590_ of the control run. The biofilm formation in the presence of the native QUE dissolved in 1% (*v*/*v*) DMSO was characterized for comparison.

#### 3.2.7. Swimming Motility and Pyocyanin Production

The plate-based assay of the swimming motility of *P. aeruginosa* PAO1 in the presence of the QUE–CHI nanoplex was characterized following the methods presented in Manner and Fallarero [18]. Briefly, 2 µL overnight inoculum *P. aeruginosa* PAO1 was spotted on a soft agar plate (0.3% *w*/*v*) containing 10 mg/mL tryptone, 5 mg/mL NaCl, and 200 µg/mL of the QUE–CHI nanoplex. The motility plate was then incubated in an upright position at 37 °C for 24 h. Afterward, the diameter of the swimming zone representing the distance traveled by the bacteria in the agar was measured. The swimming motility of the bacteria in the absence of QUE (i.e., control run) and in the presence of 200 µg/mL of the native QUE dissolved in 1% (*v*/*v*) DMSO was investigated for comparison.

The production of pyocyanin by *P. aeruginosa* PAO1 in the presence of the QUE–CHI nanoplex was quantified using four replicates following the methods presented in Ouyang et al. [17]. Briefly, an agar plate containing *P. aeruginosa* PAO1 was treated with 200 µg/mL QUE–CHI nanoplex suspension followed by 24 h incubation at 37 °C. Afterward, the pyocyanin produced was extracted by the addition of 4.5 mL chloroform to 7.5 mL cell-free filtered supernatant and re-extracted into 1.5 mL 0.2M HCl under gentle shaking until the solution turned to pink color. The amount of pyocyanin in the pink solution was then determined by a UV-vis spectrophotometer using OD_520_. The pyocyanin production in the absence of QUE was used as the control run. The pyocyanin production in the presence of the native QUE dissolved in 1% (*v*/*v*) DMSO was characterized for comparison. The cell growth was characterized by UV-vis at OD_600_.

#### 3.2.8. Statistical Analysis

All experiments were performed with a minimum of three replicates, and the results were presented as mean ± standard deviation. The statistical significance was analyzed using Student’s t-test in GraphPad Prism software (USA). All *p*-values were two-sided and considered significant at *p* ≤ 0.05 unless stated otherwise.

## 4. Conclusions

In the present work, we successfully prepared and characterized stable amorphous QUE–CHI nanoplex exhibiting a higher kinetic solubility (≈5×  higher at its peak) than the native QUE as a potential QS-inhibiting agent for inhaled bronchiectasis therapy. Unlike the other QUE solubility enhancement strategies, the QUE–CHI nanoplex preparation was simple, rapid and free of organic solvents. At its optimal preparation condition, QUE–CHI nanoplex sized around 150 nm with roughly 25% QUE payload was produced at nearly 60% QUE utilization rate and 40% overall production yield. Importantly, the QUE complexation with CHI was not found to have any adverse effect on the antibacterial and anticancer activities of QUE, hence indicating that the therapeutic activities of QUE were not jeopardized by the complexation. In comparison to the native QUE, the QUE–CHI nanoplex was demonstrated to possess a superior QS inhibition capacity in terms of suppressing the swimming motility and biofilm formation of *P. aeruginosa*, but not in suppressing the production of pyocyanin virulence factor. In addition to the higher QUE exposures afforded by the nanoplex as a result of its higher solubility, therapeutic applications using the QUE–CHI nanoplex could also benefit from the QS-inhibiting activity of CHI. The synergistic QS-inhibition effects of QUE and CHI in the nanoplex would enable us to achieve the desired therapeutic impacts at lower QUE exposures.

## Figures and Tables

**Table 1:** OD_600_ values were observed at different QUE concentrations.

QUE (µg/mL)	100	200	300	400	500
QUE–CHI nanoplex	0.504 ± 0.044	0.333 ± 0.049	0.271 ± 0.095	0.163 ± 0.149	0.048 ± 0.067
Native QUE	0.329 ± 0.051	0.301 ± 0.039	0.252 ± 0.001	0.195 ± 0.054	0.097 ± 0.029

## Data Availability

The data presented in this study are available on request from the corresponding author.

## Abbreviations

AA	Acetic acid
C_Sat_	Thermodynamic saturation solubility
CHI	Chitosan
COPD	Chronic obstructive pulmonary disease
DLS	Dynamic light scattering
DMEM	Dulbecco’s modified Eagle’s medium
DMSO	Dimethyl sulfoxide
EPS	Extracellular polymeric substance
FESEM	Field emission scanning electron microscope
FTIR	Fourier-transform infrared
HPLC	High-performance liquid chromatography
LB	Luria–Bertani
MHB	Mueller–Hinton broth
MIC	Minimum inhibitory concentration
MW	Molecular weight
OD	Optical density
PBS	Phosphate-buffered saline
PDI	Polydispersity index
PXRD	Powder X-ray diffraction
QUE	Quercetin
QS	Quorum sensing
UV-vis	Ultraviolet-visible

## Appendix A

### Appendix A.1. MIC of the Free CHI

**Table A1:** Minimum inhibitory concentration (MIC) of the free CHI.

CHI (µg/mL)	25	50	75	125	250
OD_600_	0.754 ± 0.010	0.860 ± 0.003	0.926 ± 0.008	0.048 ± 0.009	0.046 ± 0.003

### Appendix A.2. Cytotoxicity of DMSO and the Free CHI

The cytotoxicity of DMSO and the free CHI towards the A549 cells were investigated at DMSO and CHI concentrations equal to their corresponding amounts in the nanoplex (for CHI) or in the native QUE solution (for DMSO) when the QUE exposures were equal to 3, 15, and 30 µg/mL.
